# Adjuvant chemotherapy after radical cystectomy: Do all patients who need chemotherapy after surgery actually receive it?

**DOI:** 10.1097/CU9.0000000000000099

**Published:** 2022-08-02

**Authors:** Roy Croock, Jonathan Modai, Yuval Avda, Igal Shpunt, Morad Jaber, Yamit Peretz, Yaniv Shilo, Dan Leibovici

**Affiliations:** Department of Urology, Kaplan Medical Center, Rehovot, Israel

**Keywords:** Adjuvant chemotherapy, Muscle-invasive bladder cancer, Neoadjuvant chemotherapy

## Abstract

**Background:**

Compliance with the guideline recommendations for neoadjuvant chemotherapy in patients with muscle-invasive bladder cancer is incomplete. The adjuvant chemotherapy approach has the advantage of pathology-based decision-making, allowing for patient selection. In addition, radical surgery is not delayed and treatment-related toxicity does not impair surgical fitness. The proportion of patients who completed chemotherapy after cystectomy among those who were fit and in need of treatment were evaluated. The reasons for not completing adjuvant chemotherapy were determined.

**Materials and methods:**

We retrospectively evaluated all patients who had undergone radical cystectomy at our center over the last 7 years. Indications for adjuvant chemotherapy included pathological T > 2, any node+, or surgical margin involvement. Only patients who were fit for chemotherapy before surgery were included in the study.

**Results:**

Of the 52 patients with muscle-invasive bladder cancer, 14 received neoadjuvant chemotherapy or unfit for chemotherapy were excluded. Of the remaining 38 patients, 14 (37%) had bladder-confined cancers and did not require additional chemotherapy. Of the 24 patients who needed chemotherapy and were fit to receive it, 8 patients completed treatment (33%), and 3 discontinued treatment due to toxicity. Twelve patients (50%) declined chemotherapy, whereas 1 patient became unfit for chemotherapy after surgery.

**Conclusions:**

While the adjuvant chemotherapy approach could save unnecessary treatment in 37% of patients, two-thirds of those who needed chemotherapy did not complete it. Patient refusal was the primary reason for not receiving treatment.

## 1. Introduction

The current urological guidelines recommend neoadjuvant chemotherapy before radical cystectomy for all patients with muscle-invasive bladder cancer (MIBC).^[1–3]^ These recommendations stem from randomized trials showing a 5%–7% overall survival benefit in patients who received systemic chemotherapy before radical cystectomy versus cystectomy alone.^[4–7]^ Despite the survival advantage of neoadjuvant chemotherapy in patients with MIBC, there is only partial compliance with the guidelines, as many urologists initially offer radical cystectomy and decide on adjuvant therapy based on the final pathology report.^[8,9]^ Arguments supporting initial radical cystectomy include treatment-related toxicity and delay in surgery, especially among patients who are nonresponders to neoadjuvant therapy.^[10]^ In addition, up to 25% of patients with MIBC whose cancer is confined to the bladder can be cured with cystectomy alone, and additional chemotherapy may be unnecessary.^[11,12]^ An important argument against initial cystectomy is that surgery may be associated with significant morbidity, rendering some frail patients unfit for subsequent chemotherapy. The magnitude of this effect is important because if ineligibility for adjuvant chemotherapy after cystectomy is common, a significant proportion of patients requiring chemotherapy would receive suboptimal management if treated with initial cystectomy as opposed to neoadjuvant chemotherapy. In this particular case, the potential benefits of avoiding unnecessary treatment in a fraction of patients may be outweighed by the disadvantage of not providing chemotherapy to those who require it.

Our study primarily aimed to determine the percentage of patients who received adjuvant chemotherapy among those who were fit and had absolute indications for such treatment. We then clarified why the patients were not treated.

## 2. Materials and methods

Medical charts of patients who underwent radical cystectomy for MIBC at our medical center between January 2013 and April 2020 were reviewed. In this study, absolute indications for adjuvant chemotherapy were based on the final pathology and included any pathological T > 2, any N > 0, or positive surgical margins. Patients without MIBC and those who received neoadjuvant chemotherapy were excluded. Adjuvant chemotherapy consisted of 4 courses of cisplatin (70 mg/m^2^) and gemcitabine (1250 mg/m^2^). Patients who completed fewer than 3 courses of adjuvant therapy were defined as “not treated.”

The Charlson Comorbidity Index scores and 6-week postoperative kidney function (glomerular filtration rate, mL/min) were documented. Patient eligibility for systemic chemotherapy before and 6 weeks after radical cystectomy was determined. Contraindications to chemotherapy (patients unfit for chemotherapy) included a glomerular filtration rate <60mL/min, or poor performance status (European Collaboration Oncology Group score >2), significant coronary artery disease, or heart failure (NYHA class III). Patients who were considered fit for chemotherapy before surgery but developed one or more contraindications for chemotherapy 6 weeks after cystectomy were considered to have converted to being unfit as a result of the initial cystectomy. The proportion of patients with MIBC who received adjuvant chemotherapy among those who required such therapy based on their final pathology report and were defined as fit for chemotherapy before surgery was determined. Patients who were unfit for chemotherapy before cystectomy were excluded. In our study, all patients received neoadjuvant chemotherapy prior to cystectomy per the guideline medicine; however, only 8 among 46 eligible patients (17%) agreed. Among those in whom adjuvant chemotherapy was indicated but not received, the reasons for them not being treated were elucidated.

## 3. Results

**Figure 1:**
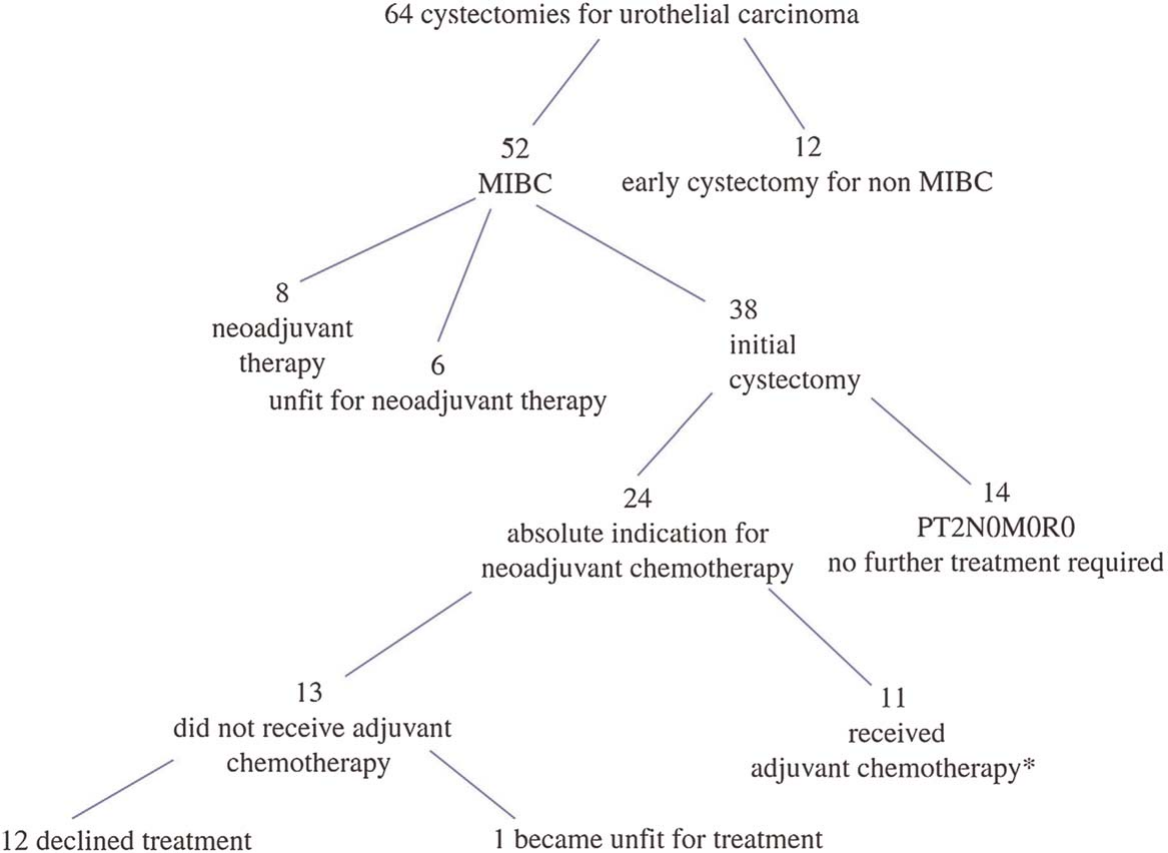
Patient flow chart. MIBC = muscle invasive bladder cancer.* Eight patients completed therapy, three patients discontinued treatment.

Sixty-four patients underwent radical cystectomy for urothelial cancer during the study period at our medical center. Of these, 52 had MIBC, and 12 underwent early cystectomy for high-risk non- MIBC. Eight patients who received neoadjuvant chemotherapy were excluded from the study. Six patients were ineligible for chemotherapy before cystectomy and were excluded from the analysis. The remaining 38 patients were included as the study participants (Fig. 1). On the basis of the final pathology report, 14 patients (37%) had bladder-confined cancer (pT2N0M0R0) and did not require adjuvant chemotherapy. Of the remaining 24 patients who were both fit for and required treatment, 11 (46%) received any number of courses of adjuvant chemotherapy; however, only 8 patients completed three or more courses of chemotherapy (Table 1). Three patients discontinued the therapy because of neutropenic fever, elevated creatinine levels, and cognitive impairment.

**Table 1:** Clinical and pathological data of the 24 patients who were fit for chemotherapy and had indications to receive such treatment.

	Did not receive chemotherapy	Received chemotherapy	*p*
Total patients, n	13	11	
Age (median), yr	67	70.5	0.21
Gender			0.8
Male	11	11	
Female	2	0	
Final pathology			0.85
T3+	10	5	
Node +	1	0	
T3+, node +	2	6	
Completed 3+ courses of adjuvant chemotherapy	NA	8 (73%)	
Charlson Comorbidity Index score			0.75
<3	5	3	
3–4	5	7	
>4	3	1	
Ileal conduit	12	10	
Neo-bladder	1	1	

All patients, excluding one who was fit for chemotherapy prior to cystectomy, remained fit for such treatment after surgery.

## 4. Discussion

The combination of radical cystectomy with systemic chemotherapy offers a 5%–7% overall survival benefit as opposed to cystectomy alone among patients with MIBC.^[4–7]^ Unfortunately, when compared with the literature on neoadjuvant chemotherapy, few studies have assessed the impact of adjuvant chemotherapy postcystectomy, and most were not randomized controlled trials. Sternberg et al. reported a 7% progression-free survival benefit with adjuvant therapy as opposed to surgery alone,^[13]^ whereas other studies suggest that adjuvant therapy is superior to neoadjuvant therapy among patients with positive lymph nodes.^[14]^

Although the combination of radical cystectomy with systemic chemotherapy has been extensively studied, in most studies, the combination has been compared with that of radical cystectomy alone. Therefore, despite the clear benefits of combination therapy, it is uncertain whether chemotherapy should be administered before or after cystectomy. Urological guidelines support upfront chemotherapy before rather than after surgery, although there is little direct evidence supporting this choice of treatment timing. A single randomized trial directly compared adjuvant versus neoadjuvant chemotherapy but failed to show an advantage over any approach.^[15]^ Therefore, it is conceivable that the addition of systemic chemotherapy indeed improves outcomes; however, its timing is of less importance.

One significant justification for adjuvant therapy over neoadjuvant therapy is that a significant percentage of patients with MIBC may be cured by radical cystectomy alone,^[12,13]^ thus precluding the need for exposure to toxic chemotherapy in this patient subset. This may have contributed to the patient’s decision not to receive neoadjuvant therapy, although the patients’ reasoning remains unclear. However, not all patients receiving combination therapy with adjuvant chemotherapy after cystectomy will complete their treatment plan. Factors such as postoperative morbidity or patient choice may preclude preplanned chemotherapy and deprive patients of optimal oncologic treatment. Our results showed that 54% of patients eligible for and requiring adjuvant therapy based on the final pathology results (pT3, N+, or R+) did not receive any chemotherapy after surgery. In addition, a few patients experienced toxicity from chemotherapy after cystectomy, rendering them unable to complete therapy. As a result, the proportion of patients who completed dual therapy among those who were fit and needed it was 33% in our report.

The dominant reason for not receiving adjuvant chemotherapy in whom it was indicated and could have received it was patient refusal, whereas surgical morbidity, rendering patients unfit for adjuvant treatment, occurred in a single patient. These findings imply that although initial cystectomy is well tolerated by most patients and offers a favorable pathology in 37% of cases, it is associated with failure to administer chemotherapy in 66% of patients requiring treatment primarily due to patient choice and not related to patient fitness or complications from surgery.

The limitations of our study are related to its retrospective design and small sample size, which ultimately limit our ability to develop a standard treatment regimen for this patient population. Additionally, patients’ choices may reflect local feelings and beliefs that are not necessarily inferable elsewhere. Furthermore, patient compliance to neoadjuvant chemotherapy was not assessed. Nevertheless, our results are significant in showing that more patients are harmed when surgery is performed initially than in those who benefit from avoiding unnecessary chemotherapy, as these patients tend to decline adjuvant chemotherapy despite the absolute indications for treatment. Improved patient compliance may be achieved if a neoadjuvant approach is adopted.

## 5. Conclusions

Our findings show that half of the patients who required chemotherapy after radical cystectomy declined such treatment. Becoming physically unfit for postoperative chemotherapy secondary to surgery is uncommon. When considering all reasons for not completing chemotherapy, only a third of the patients who needed it and were fit for treatment actually completed the entire treatment plan. These results suggest that adopting a neoadjuvant chemotherapy approach could improve the proportion of patients with MIBC receiving dual-modality treatment.

## Acknowledgments

None.

## Statement of ethics

This study received Helsinki approval study number 0092-19- KMC. According to the institutional regulations, this study did not require patient consent or institutional review board approval, for it was a retrospective study based on patients’ medical files only.

## Conflict of interest statements

The authors declare no conflicts of interest.

## Funding source

None.

## Author contributions

All authors contributed equally in this study.
